# Towards a Scottish Safe Haven Federation.

**DOI:** 10.23889/ijpds.v7i3.1842

**Published:** 2022-08-25

**Authors:** Mark Drakesmith, Gemma Hobson, Gareth John, Emily Stegall, Ashley Gould, John Parkinson, Daniel Rhys Thomas

**Affiliations:** 1 Public Health Wales; 2 Digital Health and Care Wales; 3 University of Glasgow/Scottish Centre for Administrative Data Research (SCADR)

## Objectives

To design and test a method to assess whether test events were associated with an increase in risk of confirmed COVID-19, in order to inform policy on the safe re-introduction of spectator events following decreasing incidence of COVID-19 and relaxing of restrictions.

## Approach

We designed a cohort study to measure relative risk of confirmed COVID-19 in those attending two large sporting events in South Wales during May-June 2021. First, we linked ticketing information to records on the Welsh Demographic Service (WDS) and identified NHS numbers for attendees. We then linked attendees to routine SARS-CoV-2 test data to calculate incidence rates in people attending each event for a fourteen days period following the event. We selected a comparison cohort from WDS for each event, individually matched by age band, gender and locality of residence. Risk ratios were then computed for the two events.

## Results

We successfully assigned NHS numbers to 91% and 84% of people attending the two events, respectively. Other identifiers were available for the remainder. Only a small number of attendees (<10) had a record of confirmed COVID-19 following attendance at each event (14 day cumulative incidence: 36 and 26 per 100,000, respectively). Background incidences in Wales over the same periods were 22 and 61 per 100,000, respectively. There was no evidence of significantly increased risk of COVID-19 at either event (event 1: 3.00 (0.18-47.9), p=0.50, event 2: 0.30 (0.04-2.34), p= 0.23). However, event 1, which didn’t include pre-event testing in their mitigations, had a higher risk ratio (>1) than event 2 (<1), which did include pre-event testing.

## Conclusion

We demonstrate the potential for data linkage to inform COVID-19 policy regarding sporting events. At that point in the epidemic, there was no evidence that attending large sporting events increased risk of COVID-19. However, these events took place between epidemic waves when background incidence and testing rate was low.

